# Serum Vitamin D and Pyridinoline Cross-Linked Carboxyterminal Telopeptide of Type I Collagen in Patients with Ankylosing Spondylitis

**DOI:** 10.1155/2015/543806

**Published:** 2015-07-26

**Authors:** Pingping Zhang, Qiuxia Li, Qiujing Wei, Zetao Liao, Zhiming Lin, Linkai Fang, Jieruo Gu

**Affiliations:** Department of Rheumatology, The Third Affiliated Hospital of Sun Yat-sen University, Tianhe Road 600, Guangzhou 510630, China

## Abstract

*Objective*. To assess the serum vitamin D and ICTP levels in patients with ankylosing spondylitis (AS) and investigate their relationship with disease activity and bone mineral density (BMD). *Method*. 150 patients and 168 controls were included. Serum 25(OH)D, ICTP, C-reaction protein (CRP), Bath AS Disease Activity Index (BASDAI), Bath AS Functional Index (BASFI), and Hip BMD were assessed in patients. 25(OH)D and ICTP were detected in controls. *Results*. The serum 25(OH)D in AS was 57.92 ± 24.42 nmol/L, significantly lower than controls (91.24 ± 42.02 nmol/L). Serum ICTP in AS was 5.72 ± 3.88 ug/L, significantly higher than controls (3.69 ± 1.26 ug/L). ICTP level was higher in men than in women patients (6.07 ± 4.05 versus 3.84 ± 1.96 ug/L, *P* ≤ 0.01); it was also higher in JAS than in AAS (9.52 ± 3.79 versus 5.27 ± 3.65 ug/L, *P* ≤ 0.01). Furthermore, 25(OH)D was negatively correlated with ICTP. Low 25(OH)D and high ICTP were one of the reasons of AS patients' low hip BMD. Besides, a significant relationship was found between serum ICTP and CRP. *Conclusion*. There was a high incidence of vitamin D inadequacy in AS. Serum ICTP level was elevated in AS, especially in JAS and male patients. 25(OH)D and ICTP seem to be valuable markers to detect bone loss in AS.

## 1. Introduction

Ankylosing spondylitis (AS) is a chronic inflammatory disease that primarily affects the axial skeleton. Although it is characterized by new bone formation, which leads to the formation of syndesmophytes and ankylosis of the spine and sacroiliac joints [1], osteoporosis is also a well-recognized complication of AS. It is also the main reason of the patients' spinal deformity, bone pain, and disability.

It is well-known that, as a secosteroid hormone, vitamin D is crucial in maintaining bone health. Many studies have shown that vitamin D participates in the regulation of the body's immune system, so it is helpful to obtain adequate vitamin D in the patients with rheumatic diseases. The pyridinoline cross-linked carboxyterminal telopeptide of type I collagen (ICTP) is a specific component of type I collagen, only generated from damaged mature bone matrix, and can represent a sensitive indicator of bone resorption in vivo. Till now, lots of researches on ICTP have been done worldwide, but most of them were about its relationship with malignant bone metastases. It was reported that there was a positive correlation between serum ICTP activity and pathological bone resorption [2, 3]. Unexpectedly, few studies were carried out on serum ICTP level in patients with AS and other rheumatic diseases.

The aim of this study was to elucidate the alteration of serum ICTP and vitamin D level in patients with AS and to further investigate the relations between ICTP, vitamin D, disease activity (CRP, BASDAI), and BMD in these patients.

## 2. Methods

### 2.1. Patients

From June 2012 to April 2013, 150 AS patients, from both the out-patients and in-patients registered in the Rheumatology Department of the Third Affiliated Hospital, were included in this study. The age ranged from 18 years to 50 years, with a mean disease duration of 8.4 years. All the patients fulfilled the modified New York criteria for AS [4]. Patients whose onset age was less than 16 years were named juvenile ankylosing spondylitis (JAS), and patients whose onset age was less than 16 years were named adult ankylosing spondylitis (AAS). 168 healthy controls with age and gender matched who visited our hospital in the same period were enrolled in this study.

Patients with the concomitant presence of inflammatory bowel disease, chronic renal or hepatic disease, diabetes mellitus, parathyroid or thyroid disease, recent fractures, malnutrition, or drug intake affecting bone metabolism (bisphosphonates, glucocorticoids, anticonvulsants, coumarin derivatives, or diuretics) were excluded, and postmenopausal, pregnant, and breast-feeding women were also excluded in this research. The study was approved by the local ethical committee, and all patients included in our study were provided with written informed consent to participate in this study. All the patients had received conventional treatment which included nonsteroidal anti-inflammatory drugs (NSAIDs) and/or sulfasalazine, but no one received anti-tumor necrosis factor alpha(anti-TNF*α*) drugs. Moreover, the patients had to be on stable doses of NSAIDs, sulfasalazine, and calcium and vitamin D supplements for at least 3 months before the assessment.

### 2.2. Assessment

Laboratory assessment included complete blood count, C-reactive protein (CRP), liver and kidney function tests, HLA-B27, and serum levels of 25(OH)D and ICTP. The blood specimens of all the patients were taken after an overnight fasting. CRP was measured using the nephelometric method, and ELISA was used to detect serum HLA-B27, 25(OH)D, and ITCP. Vitamin D deficiency was defined as 25(OH)D serum level less than 12 ng/mL (50 nmol/L), vitamin D insufficiency as 25(OH)D at a level of 12–32 ng/mL (50–80 nmol/L), and vitamin D sufficiency at a level higher than 32 ng/mL (80 nmol/L) [5, 6].

Clinical assessment included collection of demographic data, disease duration, medication history (NSAIDS, sulfasalazine, and calcium and vitamin D supplements), and visual analogue scale (VAS) of patients assessment of pain and disease activity. Disease activity was evaluated by ESR, CRP, and Bath Ankylosing Spondylitis Disease Activity Index (BASDAI) [7, 8]. Physical function was assessed by Bath Ankylosing Spondylitis Functional Index (BASFI; on a scale of 0–10) [9]. BMD of lumbar spine (anterior-posterior projection at L1–L4) and hip (total proximal femur) were monitored by dual-energy X-ray absorptiometry (DXA). According to the World Health Organization (WHO) classification, osteopenia was defined as a *T*-score between −1SD and −2.5SD, and osteoporosis as a *T*-score < −2.5SD [10].

### 2.3. Data Analysis

Statistical analysis was performed with SPSS 20.0 software. In independent-sample *t*-test, the patients were divided into groups according to gender, age, disease duration, 25(OH)D, CRP, HLA, and BASDAI. In the multiple regression analysis, when a parameter is a dependent variable and the others are all the variables, regression analysis was performed with SPSS 20.0 software, and we use the method of “stepwise” to bring in variables (stepping method criteria: Entry 0.05, Removal 0.10).

## 3. Results

### 3.1. Geographic Data

150 AS patients involved 127 males and 23 females (Male : female = 5.52 : 1) and the mean age of the patients was 29.83 years (SD ±8.94); 168 controls included 128 males and 40 females (male : female = 3.20 : 1), and mean age of the controls was 31.83 years (SD ± 9.97). The differences of age and gender between AS group and healthy control subjects were not significant (*P* > 0.05). Mean level of 25(OH)D in control group was 91.24 nmol/L (SD ± 42.02), and in AS group it was 57.92 nmol/L (SD ± 24.42), which was significantly lower than the healthy controls (*P* < 0.01). Mean level of ICTP in controls and AS groups was 3.69 ug/L (SD ± 1.26) and 5.72 ug/L (SD ± 3.88), respectively, and the ICTP level was elevated significantly (*P* < 0.01) in patients with AS (Table 1).

### 3.2. Characteristics of the Study Population

The AS patients were divided into different groups according to gender, age, disease duration, HLA, CRP, 25(OH)D, and BASDAI, and the comparisons were as follows (Table 2). ICTP level was found to be higher in male and JAS patients than in female and AAS patients (*P* < 0.01), when comparing to the other groups. No differences were found in 25(OH)D, ICTP level, and hip BMD *T*-score in groups which were divided according to disease duration, CRP, and HLA-B27 (*P* = NS). Patients with lower 25(OH)D level (<50 nmol/L) had higher ICTP and lower hip BMD *T*-score (*P* < 0.01). Patients with higher BASDAI (>4) had lower lumbar spine and hip BMD *T*-score (*P* < 0.01).

### 3.3. Association of Different Clinical Biochemical Indexes and BMD

By partial correlation analysis, we found some correlations in different clinical biochemical indexes and BMD. Serum levels of 25(OH)D were inversely associated with ICTP (*P* < 0.01) and disease duration (*P* < 0.05), and there was a positive association between 25(OH)D level and hip BMD *T*-scores (*P* < 0.05). Serum levels of ICTP were positively associated with CRP but were inversely associated with hip BMD *T*-score. Unexpectedly, no association was found between ICTP and disease duration, BASDAI, BASFI, and lumbar spine BMD *T*-score. Moreover, BASDAI was found to be negatively associated with lumbar spine and hip BMD *T*-score (*P* < 0.05). CRP and disease duration were found negatively associated with hip BMD *T*-score (*P* < 0.05) but not with lumbar spine BMD *T*-score.

### 3.4. Multiple Linear Regression Model Analysis for 150 AS Patients

A multiple regression model was built to assess how biochemical index, disease activity, and clinical assessments could predict the variation of hip BMD in AS patients. ICTP, BASDAI, disease duration, and 25(OH)D contributed independently and significantly to the hip BMD *T*-score (*P* < 0.05) (Table 3). Similarly, another multiple regression model analysis illuminated that ICTP contributed independently and significantly to the 25(OH)D level in AS (*P* < 0.01) (Table 4). Meanwhile, 25(OH)D and CRP contributed independently and significantly to the serum ICTP level (*P* < 0.05) (Table 5).

## 4. Discussion

Vitamin D is a secosteroid hormone which is produced in the skin under adequate exposure to sunlight or obtained from the diet. It is metabolized by a hepatic 25-hydroxylase into 25-hydroxyvitamin D (25(OH)D) and by a renal 1 alpha-hydroxylase into the vitamin D hormone calcitriol. Due to its stability and relatively long half-life, the serum 25(OH)D level is the best indicator to assess vitamin D situation in the body [11]. Vitamin D has functions beyond calcium, phosphorus, and bone, and plenty of evidences have shown that vitamin D participates in the regulation of the body's immune system [12, 13]. Deluca et al. reported that vitamin D was extremely effective in blocking the development of autoimmune disorder, and vitamin D deficiency could accelerate the appearance of symptoms and increased the severity of rheumatic disease.

Previous studies have showed the relation between a biochemical marker of type I collagen degradation (urinary CTX-I, reflecting bone resorption) and lower BMD at the hip [1, 14–16]. These studies also demonstrated that higher serum levels of bone resorption markers are associated with bone loss in AS patients with active disease. As the specific ingredients of type I collagen, which is known as the most common protein in the skeleton, comprise about 90% of the organic matrix in bone tissue, ICTP is the degradation product of mature bone matrix but not of newly formed bone. It is a newly discovered indicator of bone resorption and has stable concentration in serum. Moreover, ICTP gives responses slowly and slightly in physiologic bone metabolismbut significantly in pathological bone resorption [2]. So, ICTP can reflect the bone transformation level and is a sensitive marker of bone resorption. In the near future, it will be most probably widely used in clinical field.

So far, there have been few studies on evaluating the vitamin D and ICTP levels and their relationship to BMD in AS patients. This study showed that patients with AS were more likely to have lower serum 25(OH)D level and higher ICTP level compared to healthy individuals, and there was a common situation of 25(OH)D lacking and bone loss in AS patients. Our study revealed that about 84% AS patients were vitamin D deficient or insufficient in 25(OH)D serum level, which meant less than 32 ng/mL (80 nmol/L), and about 46.7% patients were diagnosed with osteopenia or osteoporosis whose hip BMD *T*-score was < −1SD. The pathological mechanism may be due to the high levels of plasma TNF-*α* in AS patients, which downregulates the 24-hydroxylase activity in the vitamin D system in the kidney [17]. The absorption disturbance, caused by the intestinal vitamin D receptor defect, which had been observed in AS, may also cause a decrease in vitamin D level [18]. Unexpectedly, we also found the ICTP level was much higher in men and JAS patients, although the pathological mechanism behind that is still unknown. In this study, we also found that there was no difference of 25(OH)D, ICTP, and hip BMD *T*-score level in groups of HLA-B27 positive and negative and CRP above 6 mg/L and below 6 mg/L.

Recent studies have found low level of vitamin D in AS patients is extremely common due to high disease activity [19, 20]. But in this study, no significant association was found between 25(OH)D and disease activity in AS and our findings were consistent with other previous reports [1, 5, 21]. We also found that the ICTP level was higher in the group whose 25(OH)D level was below 50 nmol/L, and 25(OH)D was inversely associated with ICTP. Thus, our study has supported the fact about the importance of intaking adequate vitamin D for AS patient in order to avoid or alleviate osteoporosis. The pathological mechanism may be that the 25(OH)D indirectly influences the bone metabolism, or the bone resorption could lead to compensatory increasing consumption of 25(OH)D, which needs more in-depth research to verify in future.

No significant correlations were found between BMD (lumbar spine or proximal femur bone) and turnover markers (such as serum carboxyterminal cross-linked telopeptide of type I collagen (CTX), osteocalcin (OC)) in a cohort study [22] of relatively young males with AS. Since the anterior-posterior lumbar spine BMD measured by DXA can be overestimated by the presence of syndesmophytes, ligament calcifications, and fusion of facet joints in these patients [1, 23–25], we used hip BMD *T*-score for further analyzing the relevant factors of osteoporosis. While comparing different groups, we found that the hip BMD *T*-score was lower in the group whose 25(OH)D level was below 50 nmol/L than the group with level above 50 nmol/L and in group whose BASDAI was above 4.

A few studies have found increased bone turnover and disease activity and decreased vitamin D levels were associated with AS-related osteoporosis in AS patients [1, 26]. According to multiple linear regression model analysis, the present study showed 25(OH)D, ICTP, BASDAI, and disease duration were independently related to low hip BMD *T*-score, which indicated 25(OH)D and ICTP, like BASDAI and disease duration, were valuable markers to detect bone loss in AS. Our findings of ICTP being positively associated with CRP in AS patients underline the importance of ICTP as a monitoring factor for disease activity. Meanwhile, the findings of 25(OH)D being inversely associated with disease duration indicate that more attention should be given to the patients with longer disease course. Besides, CRP was found positively associated with BASFI and BASDAI, and there was also a positive association between BASFI and BASDAI. In the multiple linear regression model analysis, we found no correlation between CRP, BASFI, and hip BMD *T*-score; however, BASDAI followed by ICTP made the largest contributions to the low hip BMD *T*-score.

The main limitations of our study are the fact that it is a cross-sectional study. No investigation was made to monitor 25(OH)D level at different periods of disease, and there was no data on the radiographic aspect of the spine and its scores. Also, other bone turnover markers such as osteocalcin, procollagen type I N-terminal peptide (PINP), pyridinoline, N-telopeptide, and bone specific alkaline phosphatase (BALP) were not taken into consideration. We could not properly assess the influence of drugs intake, dietary calcium supply, and seasonal differences during the study period.

In conclusion, this study indicates that there is a high incidence of vitamin D inadequacy in AS patients. As an indicator of bone resorption, serum ICTP level was elevated in AS, especially in JAS and male patients. 25(OH)D and ICTP seem to be valuable markers to detect bone loss in AS; the serum levels of vitamin D and ICTP may play an important role in the pathophysiology of AS-related osteoporosis, which needs further research on its pathogenesis.

## Figures and Tables

**Table 1:** Comparison of indicators between AS and control groups.

Factor	AS	Controls	*P* value
*n*	150	168	
Age (years)	29.19 ± 8.94	31.83 ± 9.97	*P* = NS
Gender (male/female)	127/23	128/40	*P* = NS
25(OH)D (nmol/L)	57.92 ± 24.42	91.24 ± 42.02	*P* < 0.01
ICTP (ug/L)	5.72 ± 3.88	3.69 ± 1.26	*P* < 0.01

Note: we compared means using *t*-test and proportions using chi-square test.

25-Hydroxyvitamin D (25(OH)D); pyridinoline cross-linked carboxyterminal telopeptide of type I collagen (ICTP); ankylosing spondylitis (AS).

**Table 2:** Comparison of biochemical index and BMD in different groups (AS patients were divided into groups according to different clinical factor).

AS group	*n*	25(OH)D (nmol/L)	ICTP (ug/L)	Lumbar spine BMD (*T*-score)	Hip BMD (*T*-score)
Gender					
Male	127	56.81 ± 22.79	6.07 ± 4.05	−1.64 ± 1.24	−0.90 ± 0.94
Female	23	64.08 ± 31.90	3.84 ± 1.96^**^	−1.51 ± 1.69	−1.01 ± 0.86
Clinical type					
AAS	134	58.90 ± 25.19	5.27 ± 3.65	−1.58 ± 1.35	−0.88 ± 0.90
JAS	16	49.78 ± 14.81	9.52 ± 3.79^**^	−1.96 ± 1.03	−1.21 ± 1.08
Disease duration					
>4 y	100	58.44 ± 27.58	5.78 ± 4.05	−1.54 ± 1.36	−1.02 ± 0.91
≦4 y	50	56.88 ± 16.56	5.62 ± 3.55	−1.79 ± 1.23	−0.71 ± 0.93
HLA-B27					
Positive	129	56.98 ± 25.25	5.95 ± 4.03	−1.60 ± 1.34	−0.93 ± 0.93
Negative	21	63.72 ± 17.96	4.32 ± 2.37	−1.78 ± 1.36	−0.82 ± 0.92
CRP (mg/L)					
>6	109	58.00 ± 21.65	5.94 ± 3.96	−1.71 ± 1.40	−0.99 ± 0.94
≦6	41	57.72 ± 30.92	5.16 ± 3.66	−1.37 ± 1.03	−0.72 ± 0.88
25(OH)D (nmol/L)					
>50	91		4.75 ± 2.23	−1.53 ± 1.35	−0.75 ± 0.86
≦50	59		7.23 ± 5.21^**^	−1.77 ± 1.26	−1.18 ± 0.97^**^
BASDAI					
>4	29	50.93 ± 17.25	6.70 ± 5.47	−2.21 ± 1.42	−1.31 ± 0.86
≦4	121	59.60 ± 25.62	5.49 ± 3.38	−1.51 ± 1.27^*^	−0.82 ± 0.92^*^

Note: independent-sample *t*-test was used between groups.

Values are in means ± SD.

C-reactive protein (CRP), bone mineral density (BMD), Bath AS Disease Activity Index (BASDAI), 25-hydroxyvitamin D (25(OH)D); pyridinoline cross-linked carboxyterminal telopeptide of type I collagen (ICTP).

The comparison between the two groups.

^*∗*^Statistically significant correlation *P* < 0.05.

^*∗∗*^Statistically significant correlation *P* < 0.01.

**Table 3:** Multiple linear regression model analysis for hip BMD.

Coefficients^a^					
Model	Unstandardized coefficients		Standardized coefficients	*t*	Sig.
	* β*	Std.Error	Beta		
(Constant)	−0.523	0.265		−1.971	0.051
ICTP	−0.053	0.018	−0.220	−2.852	0.005
BASDAI	−0.106	0.035	−0.224	−3.008	0.003
Disease duration	0.033	0.012	−0.208	−2.795	0.006
25(OH)D	0.007	0.003	0.180	2.332	0.021

^a^Dependent variable: hip BMD *T*-score.

**Table 4:** Multiple linear regression model analysis for 25(OH)D.

Coefficients^b^					
Model	Unstandardized coefficients		Standardized coefficients	*t*	Sig.
	* β*	Std.Error	Beta		
(Constant)	71.315	3.552		20.077	0.000
ICTP	−1.526	0.506	−0.242	−3.017	0.003

^b^Dependent variable: 25(OH)D.

**Table 5:** Multiple linear regression model analysis for ICTP.

Coefficients^c^					
Model	Unstandardized coefficients		Standardized coefficients	*t*	Sig.
	* β*	Std.Error	Beta		
(Constant)	8.990	0.922		9.755	0.000
CRP	0.087	0.017	0.627	5.155	0.000
25(OH)D	−0.042	0.011	−0.262	−3.641	0.000

^c^Dependent variable: ICTP.
